# The Survey of Double Robertsonian Translocation 13q; 14q in the Pedigree of 44; XX Woman: A Case Report

**DOI:** 10.22088/BUMS.6.4.243

**Published:** 2017-11-29

**Authors:** Nasrin Malekpour, Seyed Mohammad Amin Kormi, Mahtab Azadbakht, Meysam Yousefi, Mohammad Hasanzadeh-Nazar Abadi

**Affiliations:** 1 *Student Research Assembly, Mashhad University of Medical Sciences, Iran.*; 2 *Cancer Genetics Research Unit, Reza Radiation Oncology Center. Mashhad, Iran.*; 3 *Department of Medical Genetics, Faculty of Medicine, Mashhad University of Medical Sciences, Mashhad, Iran.*

**Keywords:** Robertsonian translocations, aneuploidy, spontaneous abortion, abnormal karyotype, prenatal diagnosis

## Abstract

Robertsonian translocations (RBTs) are associated with an increased risk of aneuploidy. Single RBT carriers are the most common balanced rearrangements among the carrier couples with the history of spontaneous abortions. However, double Robertsonian translocations (DRBTs), in which two balanced RBTs occur simultaneously, are extremely rare conditions. A 9-year-old mentally normal girl with multiple skeletal disorders was found to carry a balanced 13/14 RBT (45, XX, t(13q; l4q)). Three generations of her family, including her parents and her maternal grandparents were investigated for cytogenetic analysis. All of them were phenotypically normal. Her mother appeared in a peculiar karyotype of 44, XX, t (13q; 14q) ×2, while her father revealed a normal karyotype 46, XY. Chromosomal constitution of her grandparents showed that both of them carried this balanced reciprocal translocation (45, XY t (13q; 14q) as well as 45, XX, t (13q;14q)). Cytogenetic evaluations on the basis G-banding technique were performed for participants. Except the 9- year-old girl, all RBT carriers in this family appeared phenotypically normal, her skeletal disorders might not be due to chromosomal rearrangement. Meanwhile, all offsprings of 44, XX woman are obligatory carriers of this translocation, and should be candidates for prenatal diagnosis (PND) or preimplantation genetic diagnosis (PGD), for their future pregnancies.

Structural chromosomal rearrangements have been reported with several different classes of events such as deletions, duplications, inversions, and translocations that encompass for ~21% of all chromosome abnormalities (1, 2). Although structural chromosome rearrangements are found in approximately 1 in 1000 live births, it is estimated that 0.2% of people carry an asymptomatic chromosomal rearrangement (3). Robertsonian translocation (RBT) is the most common form of chromosomal rearrangements, which is the joining of two telo/acrocentric chromosomes at their centromere to form a meta/submetacentric chromosome, and comprises 18% of total genetic abnormalities (4). RBTs might happen between two homologous or non-homologous acrocentric chromosomes (5).

Approximately 90% of all RBTs result in non-homologous chromosomes, which involve two different acrocentric chromosomes (6). Meanwhile, most homologous translocations are only rarely observed, with the exception of t(21q; 21q), which is found in some Down syndrome patients (7). The most common balanced RBT (75%) appears with 13q; 14q. This translocation may arise *de novo* (~50%) or be inherited (8).

Although carriers of balanced RBTs are phenotypically normal, nevertheless they can produce a significant percentage of unbalanced gametes causing early spontaneous abortions, fetal losses, mental retardation, multiple congenital anomalies, uniparental disomy, and infertility (9-12). Double Robertsonian translocation (DRBT) is a condition that two balanced RBTs occur simultaneously. Despite the relatively high incidence of RBTs, it has been reported that DRBTs are extremely rare conditions, and may be due either to inheritance or *de novo* centric fusions (9). In this study, we report a woman with peculiar karyotype 44, XX, t (13q; 14q) ×2 and also followed 3 generations of her family for understanding the origin of this phenomenon.

**Case report**

A normal female with a DRBT karyotype is reported in this study. The case is a 38 -year- old woman that had a mentally normal 9-year-old daughter with skeletal disorders including transverse growth and thickening of the bones, polydactyly of the hands and feet, and micrognathia associated with significant increase of chin protrusion. The girl was referred to the medical cytogenetic laboratory at Imam Reza Hospital affiliated with Mashhad University of Medical Sciences, for cytogenetic analysis. Chromosomal studies were performed on the basis of G-banding technique. The results indicated that the girl was a carrier of a balanced 13q; 14q RBT with chromosomal constitution 45, XX t(13q; 14q) (Figure 1), which may not be related to her disorder. Thereafter, the informed consent with the appropriate local ethics review committee approval was obtained from patient and patient’s family, karyotyping was carried out for her phenotypically normal parents. Her parents were first cousins, and their chromosomal investigation showed DRBT 13q; 14q, for her mother with chromosomal constitution 44, XX t(13q; 14q)×2 (Figure 2), and a normal karyotype 46, XY for her father (figure 3). In order to find the origin of such unusual rearrangement, chromosomal analysis for her maternal grandparents was accomplished, and results indicated that both of her grandparents were heterozygous carriers for RBT 13q; 14q, (45, XX t(13q; 14q) for grandmother and 45, XY t(13q; 14q) for grandfather) (Figures 4 and 5). They were healthy and clinical examination indicated no abnormality. Also, they had no history of spontaneous abortions or congenital disorders. **Figure 6** shows the pedigree of the studied family.

**Fig. 1:**
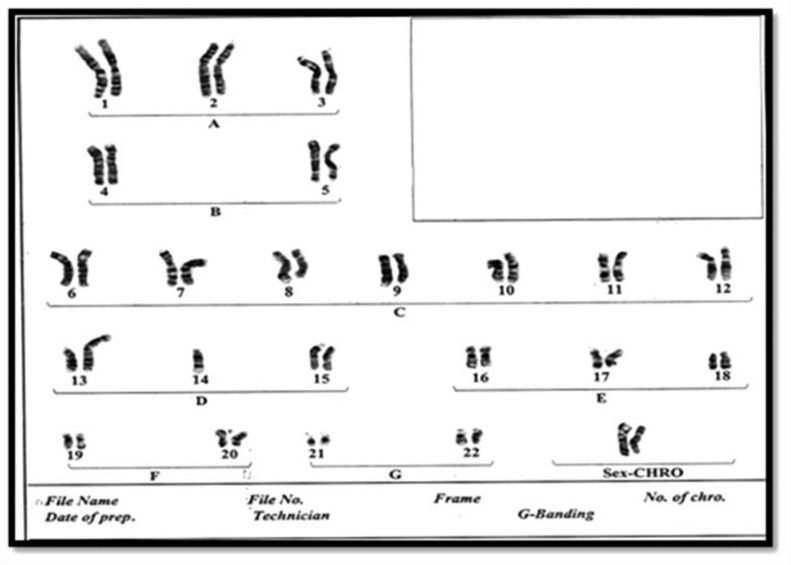
Karyotype of 9-years old daughter with balanced Robertsonian translocation between chromosomes 13 and 14, 45 XX 13q/14q

**Fig. 2:**
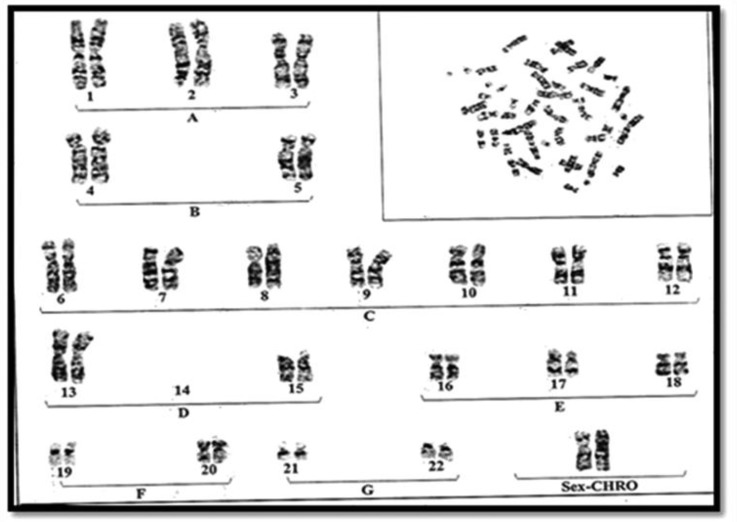
Karyotype of the mother with balanced double Robertsonian translocation between 2 chromosomes 13 and 2 chromosomes No 14, 44 XX t(13q/14q) ×2.

**Fig. 3:**
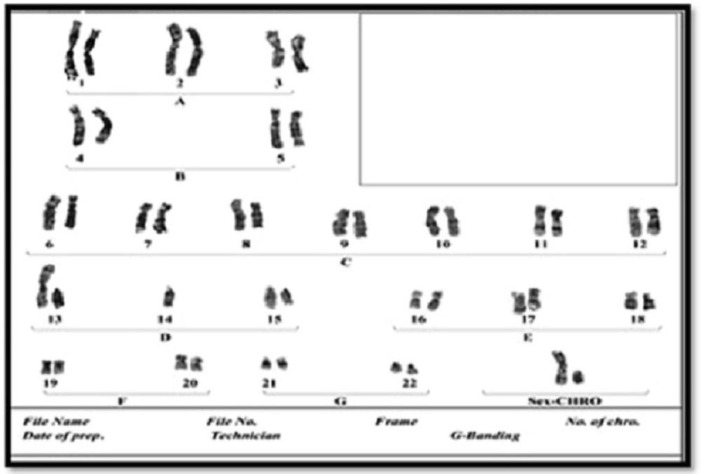
Karyotype of the father with normal karyotype 46, XY

**Fig. 4:**
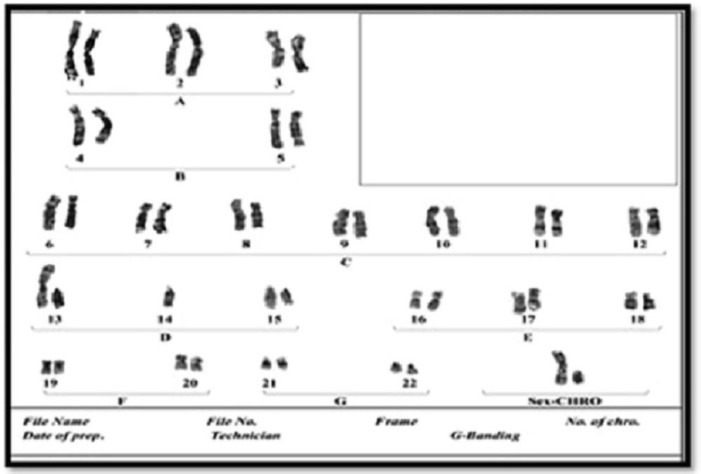
Karyotype of grandfather with Robertsonian balanced translocation between chromosomes 13 and 14, 45 XY 13q/14q

**Fig. 5:**
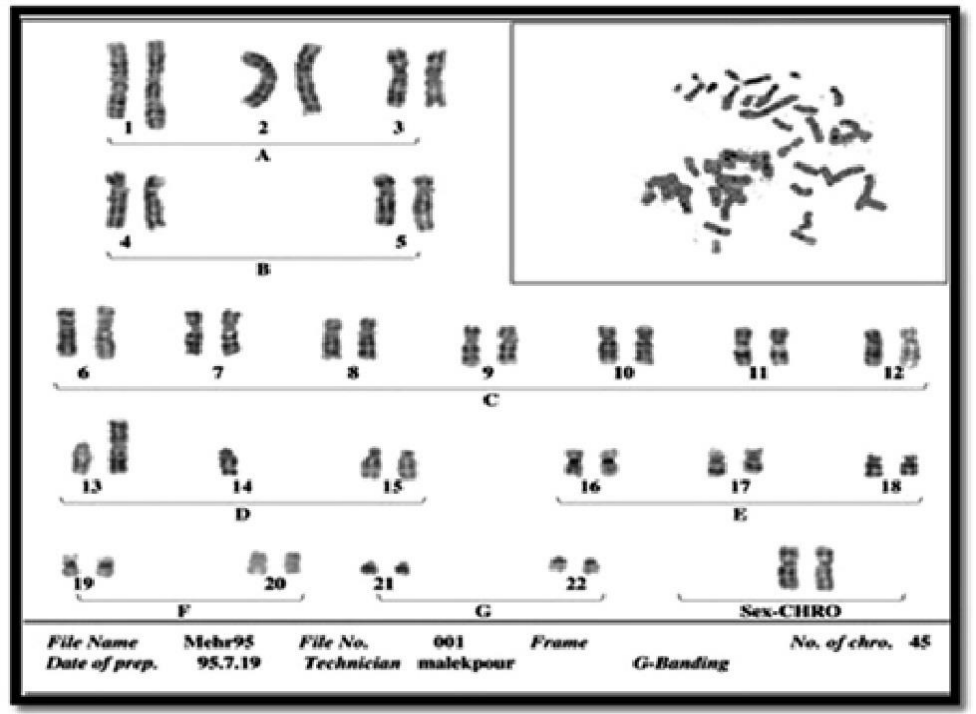
Karyotype of grandmother with balanced Robertsonian translocation between chromosome 13 and 14, 45 XX 13q/14q

## Discussion

RBTs are associated with an increased risk of aneuploidy. The single chromosomal RBT is common among carrier couples with the history of spontaneous abortion (13). Here we performed chromosomal analysis of a family with RBT, and identified a woman with DRBT. Heterozygous carriers of RBT have experienced poor fertilization outcomes. During the first meiotic division, the main risk for carriers of a balanced chromosomal rearrangement, is the production of a high proportion of unbalanced gametes (14). Therefore, they are at high risk for spontaneous abortions as well as chromosomally unbalanced offsprings (4). Hasanzadeh-Nazarabadi et al. reported a family with a history of recurrent pregnancy loss. Cytogenetic analysis indicated similar balanced RBT between chromosomes 21 and 14 in 6 members of this family (15). Wang et al. reported a girl with multiple congenital anomalies that carried a balanced 13; 14 RBT. The patient inherited both chromosomes 14 from her father and none from her mother (16). However, DRBT is an extremely rare condition. So far, a few numbers of DRBTs were reported in the world, representing the evidence for such rare condition. Dallapiccola et al. reported a couple of first cousins with a spontaneous second-month abortions. The couple were found to be heterozygous for an RBT t(14; 21) (p11; q11). First-trimester prenatal diagnosis (PND) in the third pregnancy of the mother revealed a 44, XY, t(14; 21) × 2 karyotype in fetus (17). Martinez-Castro et al. reported a normal couple who had a normal 6-year-old boy, but no other children were seen because of subfertility. The karyotype of the husband was normal and her wife, whose parents were first cousins, revealed a homozygous 13; 14 RBT (18). Rockman-Greenberg et al. reported a phenotypically normal 44 chromosomes fetus with homozygous 14; 21 translocations. One 14; 21 translocation was inherited from her father and another arose *de novo* (19).

Usually, DRBT carriers are born in families with blood-related parents similar to the present report that parent's of 44 XX female were first cousins, and each of them was heterozygous for RBT 13q; 14q which they could have been inherited from a common ancestor. Although the majority of DRBTs are healthy people with normal phenotype, and have a favorable reproductive prognosis, but all of their offsprings are carriers for RBT (8). Identification of a DRBT makes it possible to find their heterozygous offsprings who are at high risk of having children with imbalanced chromosomal rearrangements, and then PND will be strongly suggested for their offspring (17).

Notably, the reproductive history of parent's 44 XX female was free of abortion that is probably due to a few numbers of attempts to the pregnancy of this couple, because they have only 2 progenies. Meanwhile, her heterozygous daughter is candidate for PND or preimplantation genetic diagnosis (PGD) for her future pregnancies. Besides, her skeletal disorders are not related to chromosomal anomaly because her mother and her grandparents have the same translocation, and are free of skeletal problems. Therefore, her skeletal disorders might be due to a single gene disorder and not chromosomal rearrangement, and need more investigations (1-3).

This case adds further evidence that people with 44 chromosomes can be healthy and free of dysmorphic features. Identification of a DRBT makes it possible to find their heterozygous offsprings who are at risk of having children with imbalanced chromosomal rearrangements, and then preventing abnormal offspring birth.
